# Infection and Predisposition

**Published:** 1901-10-26

**Authors:** 


### Infection and Predisposition.

The pendulum of fashion has, during the past few years, swung far in the direction of depriving many of the influences to which people used to attribute the onset of tuberculosis of their significance. Much popular teaching, often of a somewhat *ad captandum* nature, has tended to undermine the importance of diathesis, facies, and predisposition in regard to tuberculosis. The germ has been placed on a high pedestal, infection has been made into a fetish, and the faiths of our fathers have been to a large extent deserted. Nevertheless artists and people of observation still hold to the "consumptive type," girls and young men are still said to be "consumptive looking," and those who, putting theory on one side, are content to watch events, find too often that these old notions turn out to be correct. The infectionists have in fact overdone it. To thinking men the widespread diffusion which is claimed for the infection of tuberculosis, and the multiplicity of the chances which we all have of coming across it at every turn, have always stood in the way of a thoroughgoing acceptance of the theories which are now so popular. In opposition to the modern explanation of the prevalence of consumption among the poor, namely that in homes of poverty infection has far more opportunities than in homes of light and air and cleanliness, they have held that there is something else, and that one great reason why consumption prevails so largely among the poor is that the conditions of their lives engrain in them a predisposition to the disease. Everything turns upon the comparative value of infection and predisposition, and now, after the violent oscillation set up by the popularising of the infection theory, we find the pendulum swinging back once more, under the influence of common sense, in the direction of predisposition. As we have just said, the widespread nature of the infection of tuberculosis makes it fairly clear that something besides exposure to it is involved in the development the disease. But when we find that not only are the germs widespread, but that a large proportion of the population are actually infected by them, and yet are able to throw off the disease, it becomes more evident than ever that it is the condition of the individual rather than the presence of the germ which decides whether a man shall or shall not become consumptive.

At the present day post mortem examinations at certain institutions are conducted in a much more complete and perfect manner than in times past, with the result that a much larger proportion of the bodies are found to show traces of having been at some time or another infected with tuberculosis than was formerly thought to be the case. When we hear that in 500 post-mortem examinations made at the Zurich Pathological Institute undoubted signs of tubercle were discovered in 97 per cent. of the bodies examined, and when we compare with that the comparatively small proportion of the population which is carried off by the disease, when in fact we find that far more people threw off the disease than ultimately died of it, we cannot resist the conclusion that the ultimate result of an infection with tuberculosis must depend far more upon the resisting power of the patient than on the presence or even on the reception of the infective material. Thus we find ourselves swinging back again to the theory of predisposition, whether inherited as a diathesis if we may venture once more to use the term, or developed as the result of the condition and accidents of life.

These good people at Zurich did not escape tuberculosis because they had the good luck not to come across the microbe, for 97 per cent. of them had actually been affected by the disease. Safety in such cases has been the result not of escaping infection but of over-mastering it. The vitality of the individual rather than the virulence of the germ is then the predominating influence. As Sir William Gowers has lately said, facts may be differently explained but they cannot be destroyed by new knowledge, and although there has come to be a strong popular impression that an inherited influence in the causation of tuberculosis has been disproved by the discovery of its dependence on organisms received from without, the facts which led to the belief in that inherited influence still remain. These facts are, he says, equally explained by the assumption that what is inherited is a relatively low power of destroying the tuberculous organisms which have gained entrance into the system.

Of course the same thing holds good in regard to the arguments which hinge upon the prevalence of phthisis among those who dwell amid insanitary conditions. It is not entirely the abundance or virulence of the germ but the lack of resistance in the individual which is at fault. This view was the keynote of much that was said by Dr. Allchin last week in his presidential address to the Medical Society; and so we see the pendulum swing back again towards its old position. Towards, but never quite to it; for new knowledge must always leave its mark, and make a permanent indent upon our conception of things.

